# Accelerated workflow for primary jaw reconstruction with microvascular fibula graft

**DOI:** 10.1186/s41205-017-0010-7

**Published:** 2017-02-14

**Authors:** Elisabeth Goetze, Matthias Gielisch, Maximilian Moergel, Bilal Al-Nawas

**Affiliations:** Department of Oral and Maxillofacial Surgery, University of Mainz, Medical Center, Augustusplatz 2, D-55131 Mainz, Rheinland-Pfalz Germany

**Keywords:** Head and neck cancer, Reconstruction, Microvascular flap, Fibula, CAD CAM, Computer aided

## Abstract

**Introduction:**

Major facial defects due to cancer or deformities can be reconstructed through microvascular osteocutaneous flaps. Hereby CAD/CAM workflows offer a possibility to optimize reconstruct and reduce surgical time. We present a retrospectiv observational study regarding the developement of an in-house workflow allowing an accelerated CAD/CAM fibula reconstruction without outsourcing.

**Case description:**

Workflow includes data acquisition through computertomography of head and legs, segmentation of the data and virtual surgery. The virtual surgery was transferred into surgical guides and prebent osteosynthesis plate. Those were sterilized and used in surgery.

**Evaluation:**

The workflow was used in 30 cases. Minimum planning period took 4 days from CT to surgery, average time was 8 days. Planning could be transferred to surgery every time. Intraoperative complications regarding osteotomy, assembly and fixation did not occur.

**Discussion/Conclusion:**

An in-house workflow for CAD/CAM fibula reconstruction is feasible within a few days providing an accelerated procedure even in urgent cases.

## Background

Advanced tumors or progressive chronic inflammation of the jaws frequently require segmental resection. Thereafter reconstruction by free microvascular bone transfer represents nowadays the method of choice in patients with acceptable health status [1–4]. For reconstruction of the upper and particularly the lower jaw the microvascular fibula flap is mostly utilized for extended bone defects and regularly allows integration of a skin paddle p [5, 6]. The basic concept in raising free fibula flaps was first described by Taylor in 1975 but has evolved in parts over the last decade [2, 7]. Surgery can be supported by computer aided design (CAD) based planning and preoperative manufacturing (computer aided manufacture, CAM) of surgical templates [7–9]. A CAD/CAM workflow allows preoperative definition of cutting paths and angles at the resection site, modeling of the graft as well as the shape of the osteosynthesis material resulting in an easy composable and placeable reconstruct [7]. The overall assembly time consisting in intraoperative cutting, positioning and refinement of the graft is reduced by the CAD/CAM workflow [7, 10], thereby substantially reducing risks concomitant with long-time surgery [11–13]. Last but not least integrated CAD/CAM workflow may improve the esthetic and functional outcome by optimizing position and contour of the reconstruct [7, 14].

The process of CAD/CAM planning can involve a commercial platform or be done by the clinic itself. Commercial solutions require communication between medical engineers and clinician and external logistic pathways. Exact information about average delivery time is not documented but the workflow results in a planning period of several weeks. Rustenmeyer for instance states a planning period of 2–4 weeks [15]. For in-house logarithms necessary planning time is not stated to date. Any delay in treatment may interfere with vital structures in extended malignancies or in tumor disease with rapid progression. Thus, the preoperative planning interval has to be as possibly short to provide a sufficient application of a CAD/CAM procedure. CAD/CAM procedures are described both primarily and secondarily [1, 16] but primary reconstruction in time-critical cases is not done routinely [7, 16]. Mazzoni et al [17] state that CAD/CAM procedure and surgical application should be minimized to an interval of 2 weeks.

The aim of the present retrospective observational study is to describe the development of an in-house workflow with reduced planning time and thereby allowing CAD/CAM based jaw reconstruction through microvascular fibula graft even in urgent cases.

## Case description

Retrospective analysis was done for 30 patients, the case of one patient is illustrated as full workflow. All patients gave written consent into the procedure and use of their data. For the case report the patient gave written consent in the publication of his pictures. The need of ethics approval was waived by the Ethics Commission of the State Chamber of Medicine in Rhineland-Pfalz according to Berufsordnung § 15 and Landeskrankenhausgesetz § 36 und § 37.

### Workflow

In patients diagnosed with need for jaw reconstruction by microvascular bone grafting careful evaluation of donor and recipient vessels was performed. This included a computed tomography (CT) based angiography of the lower limbs, CT with contrast agent of the head and neck region and B-mode and Doppler ultrasound of cervical and cutaneous perforator vessels of the donor site. If the diagnostic procedure revealed sufficient vessel supply the patient was enrolled for further CAD/CAM preoperative planning.

**Fig. 1:**
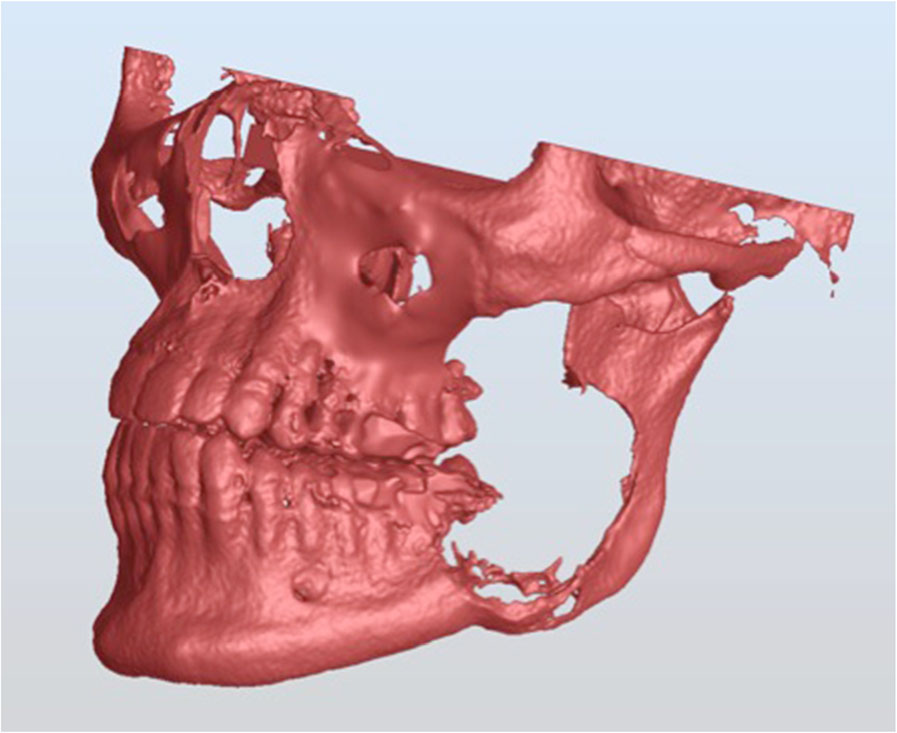
Virtual segmentation of skull and mandible (presentation in PlastyCAD®) with a defect in the left mandibular angle due to ameloblastoma

**Fig. 2:**
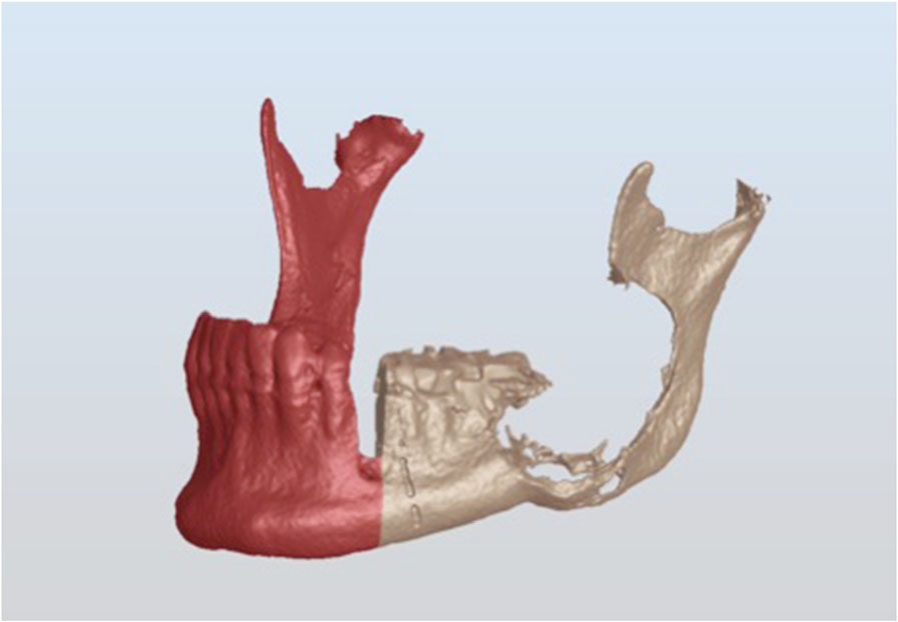
Virtual osteotomie of jaw (PlastyCAD®)

**Fig. 3:**
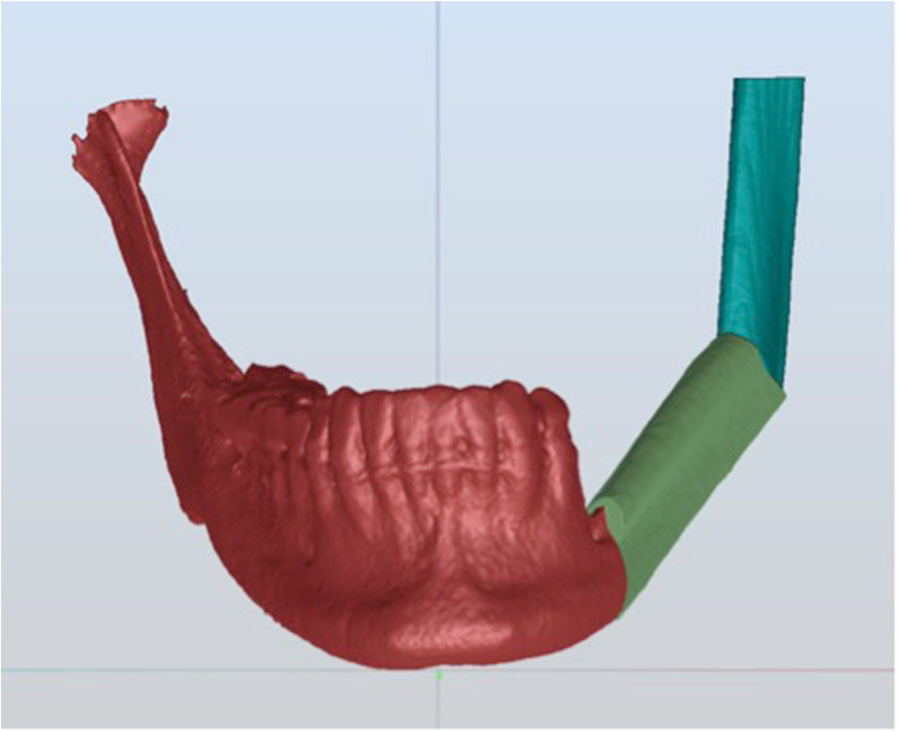
Virtual jaw reconstruction with fibula graft (PlastyCAD®)

Early planning routine consisted of a virtual and laboratory part. During the virtual session the DICOM data from the skull and lower leg were obtained and segmented (Fig. 1) using Simplant O&O® software (Materialise, Leuven, Belgium). Hereby, the resection and the reconstruction of the jaw was virtually performed (Figs. 2 and 3). In case of need the orientation of the bone segmentation took the perforator vessels of the skin flap into account. Osteotomy lines where set and the final angulations of the graft segments documented.

Since the software does not allow data export in a printable way such as stl-files (standard tesslation language) or other 3D data the planning was documented and re-performed in a laboratory model surgery. Through the laboratory model surgery templates and a pre-bent osteosynthesis plate were manufactured.

For this purpose the DICOM data was segmented into printable stl.-files via OsiriX imaging software (64bit, Version 6.0) and post-processed in NetFabb Basic (Version 5.2.1; netfabb GmbH, Lupburg, Germany) and MeshLab (Version V1.3.3; supported by 3D-CoForm project). The data was used to print a respective 3D model of the head and the bone graft. A photopolymer printer system EDEN260V (Stratasys, Eden Prairie/Minneapoulis, USA) allowed time saving printing of 3D models inside the clinic.

Once models were finished they were brought to the laboratory and a model surgery converting the virtual planning into model was done. Using this surgical templates for resection and graft osteotomies (length, angulations) as well as the bending of an osteosynthesis plate (Medartis®, Tri-Lock-System, Basel, Switzerland) for graft retention were performed.

Lately the workflow was converted into a complete digital workflow. CAD planning was performed with PlastyCAD® (3iemme, Cantù, Italy). The software allows a free export of stl-files.

**Fig. 4:**
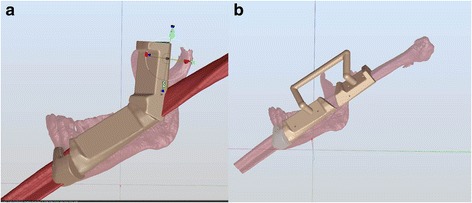
**a b** surgical guides for resection and fibula harvesting (PlastyCAD®)

After segmentation of 3D models of the skull (Fig. 1) via OsiriX, PlastyCAD® was used for simulation of the resection (Fig. 2) and reconstruct (Fig. 3). Thereafter surgical guides were designed (Fig. 4 a/b) and exported in stl. Those were printed on photopolymer printer (Material: MED610; Printer: EDEN 260 V, Stratasys®, Eden Prairie/Minneapoulis, USA).

Planning was conducted by one surgical resident under supervision of the senior residents for resection margins and final acceptance of planning. Regarding the learning process an assimilation was noticeable, but due to the different software possibilities used at the beginning a clear learning curve did not show in measurable terms. First plannings – including the laboratory part took 10 h in complete while the last semi-CAD plannings took around 5 h. The planning time thus became noticeable shorter, even so the assumption has to be made that complexity and especially the number of planned segments and necessary reposition of any kind would prolong planning and planning time thus is not only a product of experience.

**Fig. 5:**
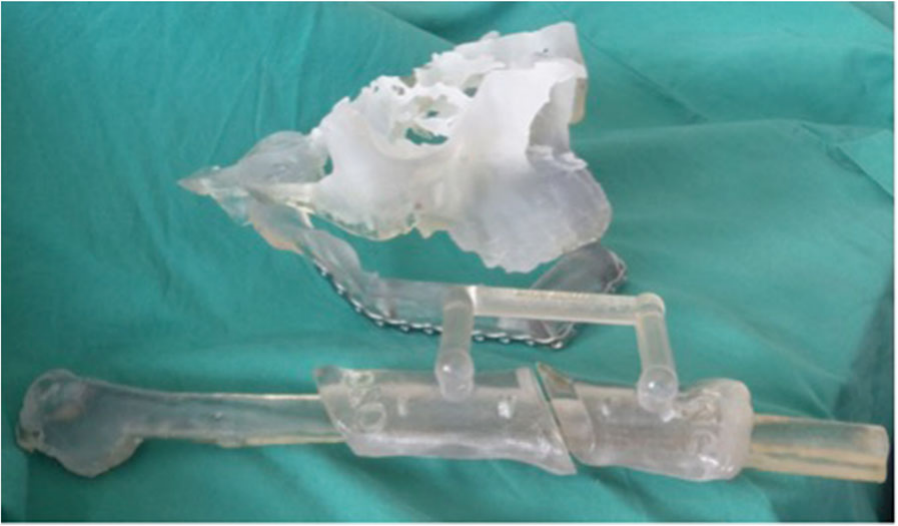
3D printed reconstruction with surgical splint and adjusted osteosynthesis plate (Medartis, Basel, Switzerland)

**Fig. 6:**
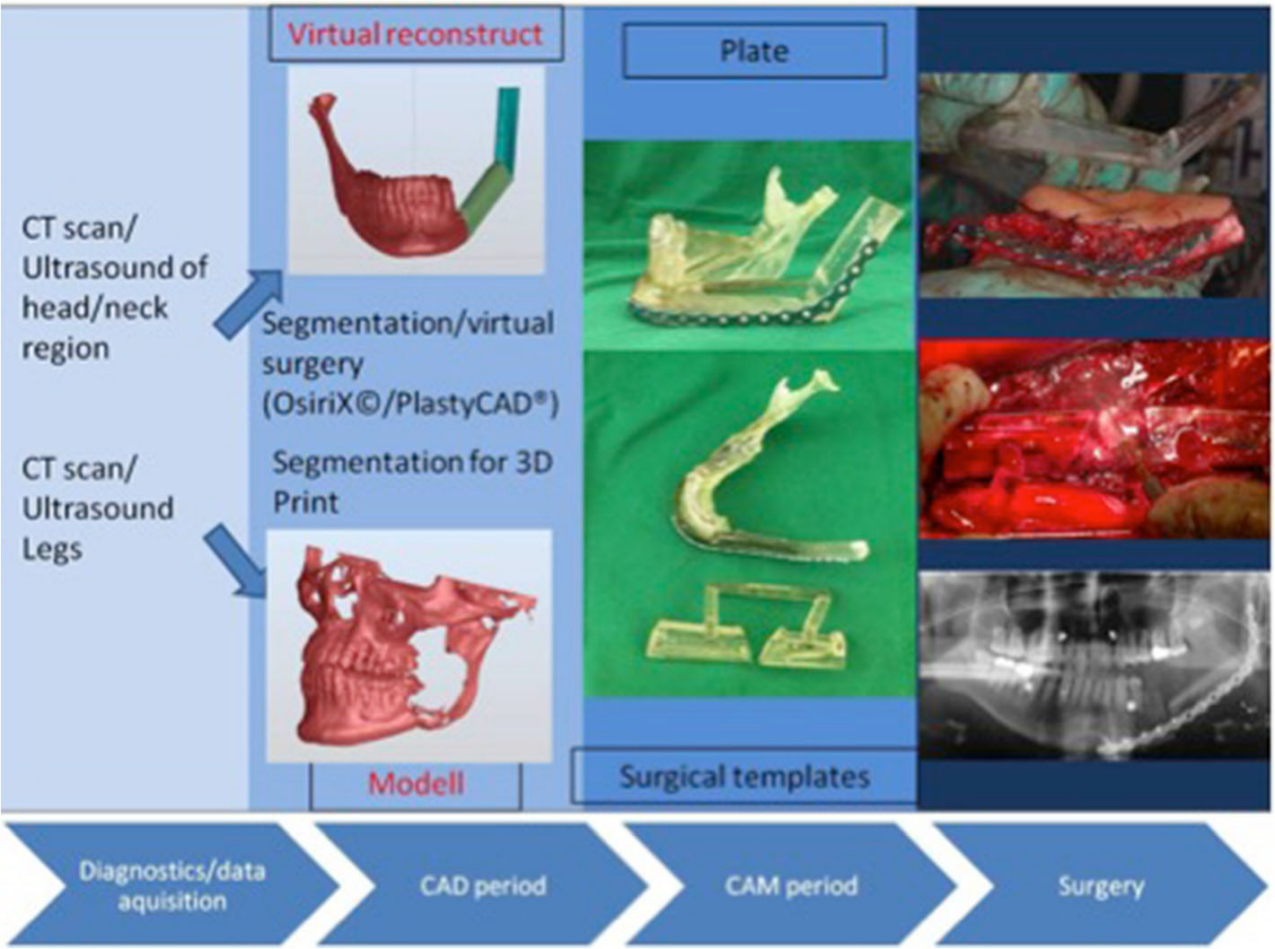
CAD/CAM planning of fibula graft, workflow

Surgery templates and pre-bent plate (Fig. 5) were autoclaved and used in surgery. The patient model lay on site for reference. The complete workflow is summarized in Fig. 6.

### Case

**Fig. 7:**
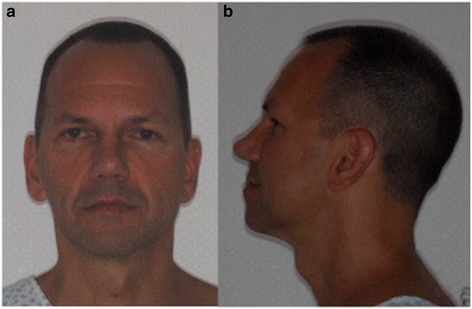
**a b** preoperative appearance of patient

**Fig. 8:**
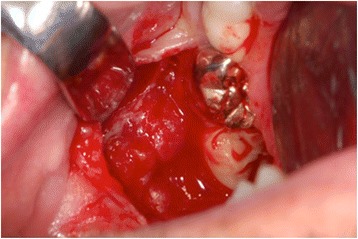
Intraoperative appearance of the tumor of the left jaw

**Fig. 9:**
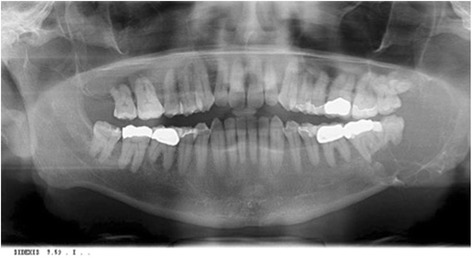
Preoperative panorama X-ray of the jaws with an erosive bone defect of the left mandible

**Fig. 10:**
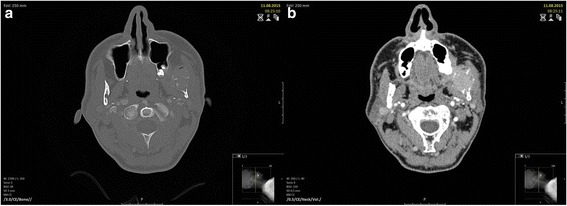
**a b** preoperative CT scan (soft tissue and bone window) showing the tumouros lesion with a bone defect in the left mandible

**Fig. 11:**
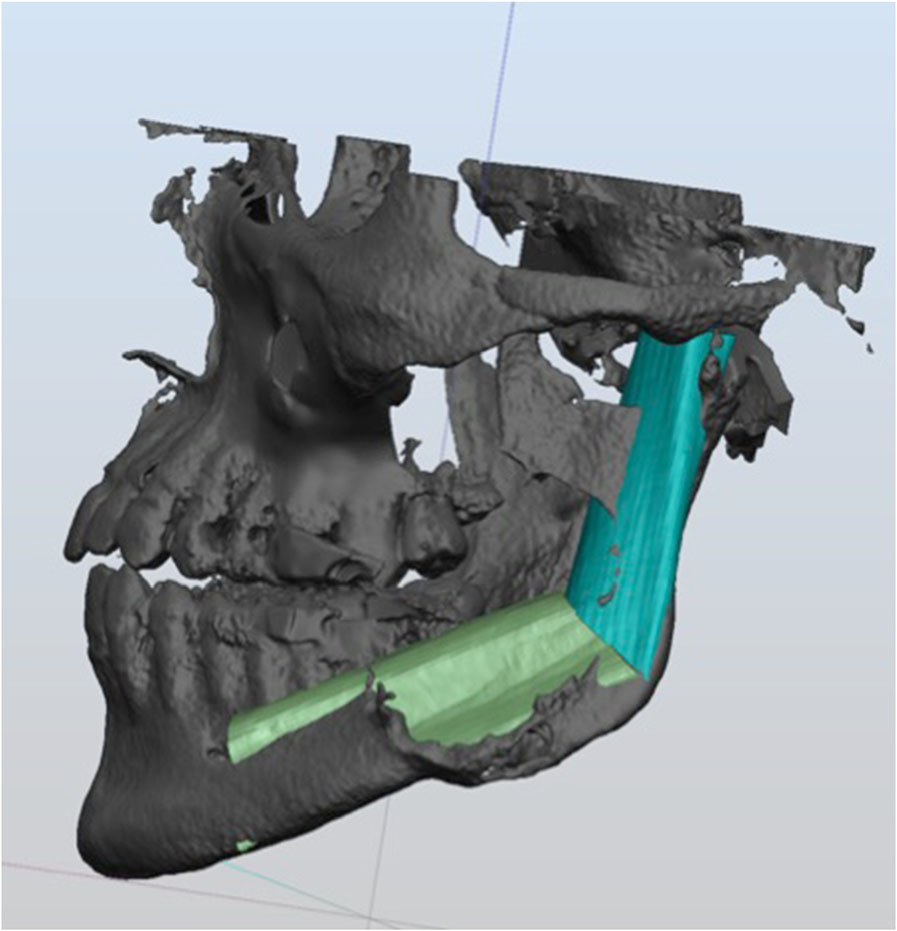
Virtual planning of resection and fibula reconstruction

**Fig. 12:**
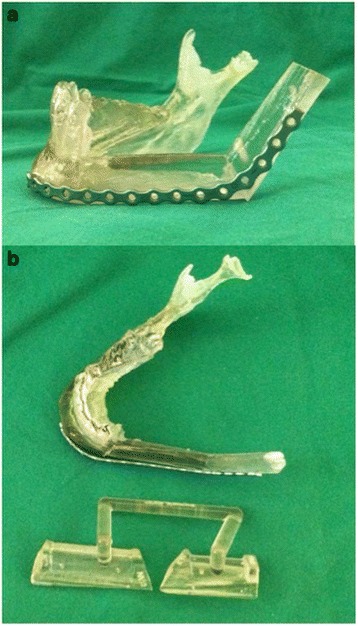
**a b** surgical model, bent osteosynthesis plate and surgical template of resection and fibula reconstruction

**Fig. 13:**
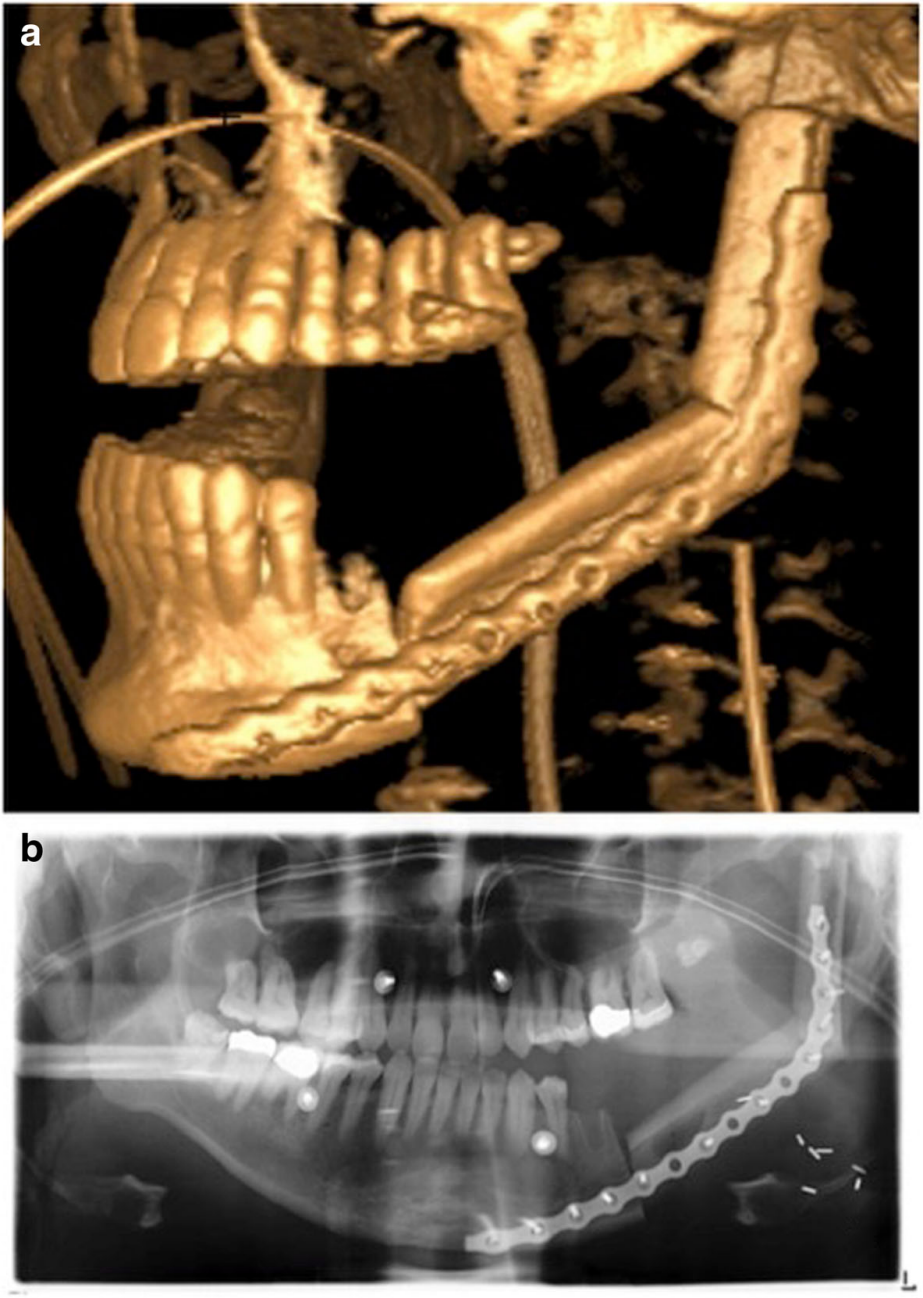
**a b** postoperative 3D reconstruction of the fibula graft (1 day after surgery) and panoramic x-ray (7 days after surgery)

**Fig. 14:**
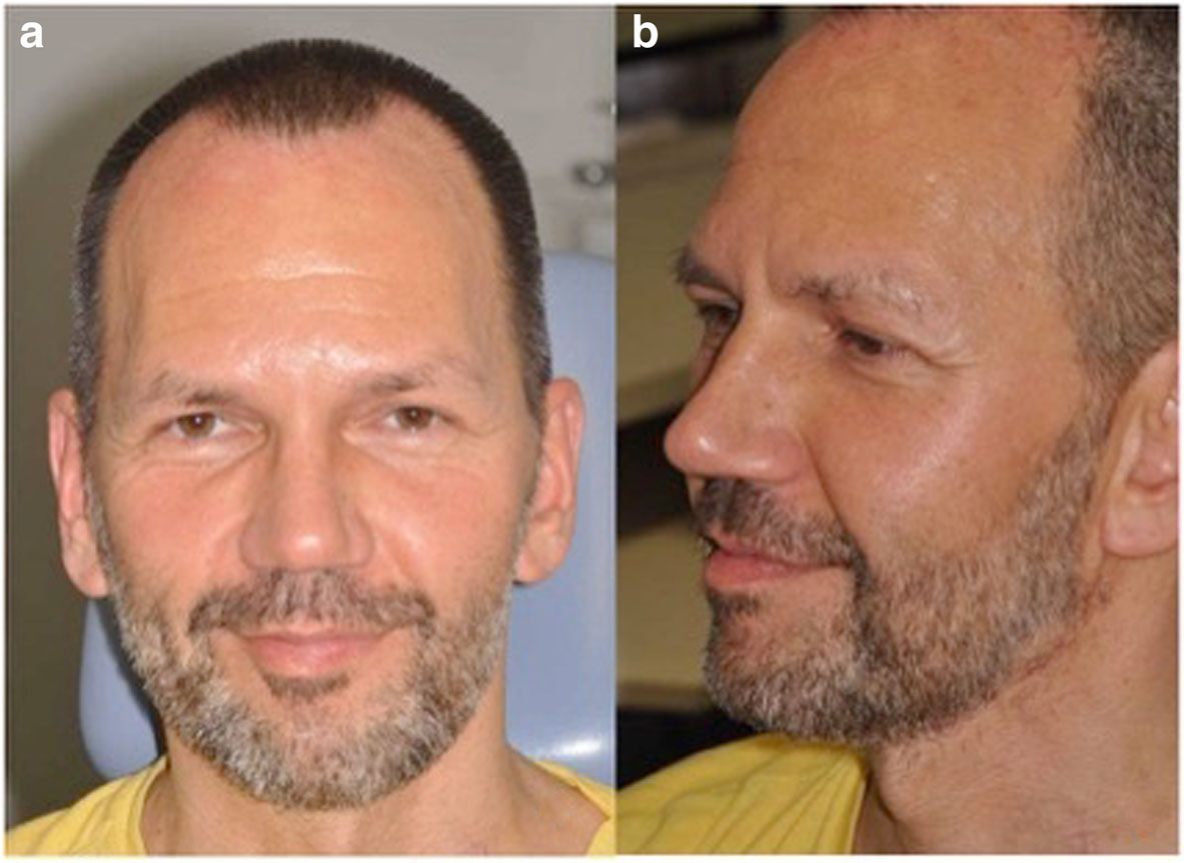
**a b** clinical appearance 3 month after surgery

The workflow (Fig. 6) is illustrated in case of a 50 year old patient (Fig. 7 a/b) treated August 2015 in our clinical department with ameloblastoma with soft tissue involvement (Figs. 8, 9 and 10) of the lower jaw. Malignancy could not be ruled out completely before surgery. Radiologic data showed suspect lymph nodes. CAD/CAM planning (Figs. 11 and 12a/b) was performed in 4 days and the patient underwent resection from the ascending ramus and the condyle as well as selective neck dissection of the ipsilateral side was performed in curative intention [18]. Histological tumor classification of the resection specimen revealed a plexiform ameloblastoma classified pT2 pN0 analogous to common neck tumor classification [19]. Postoperative X-ray showed sufficient jaw reconstruction (Fig. 13 a/b). At first swelling caused a side open bite which could be corrected through intermaxillary fixation. Until now the patient is tumor free and the clinical course was free of complications. The patient is content with his postoperative appearance (Fig. 14). Prosthetic rehabilitation is in progress.

## Evaluation

The workflow was applied in 30 cases for primary and secondary reconstruction in the time from January 2014 to January 2016. The gender distribution was 1:2 (female:male). Average age was 50 years (50 ± 17). Eight patients underwent secondary reconstruction after a tumor free interval of 1–6 years, all other patients were primarily reconstructed with tumor resection during the same surgery. All patients were reconstructed with a free microvascular anastomized fibula bone graft. Two patients did not require skin graft, 19 patients had intraoral, six patients extraoral and three patients combined intra-/extraoral skin grafts.

All patients received a probe biopsy beforehand the main procedure. The main diagnosis was oral squamous cell carcinoma (*n* = 20; primary carcinoma *n* = 14; recurrent carcinoma *n* = 6), followed by sarcoma (*n* = 4), ameloblastoma (*n* = 2), adenocystic cell carcinoma (*n* = 1) and osteoradionecrosis (*n* = 1). Twelve patients had undergone irradiation or radiochemo therapy before surgery through neoadjuvant treatment or therapy of former malignancies. Twenty patients had anamnesis of nicotine consumption. Seven patients had positive anamnesis for arteriosclerotic disease.

Planning could be applied successfully in all cases. Osteotomy and assembly time did not exert 1 h in all cases. Over all flap survival was 93%. Patient survival was 90% (*n* = 3; none sooner than 3 month after surgery). Death was caused by cardiac arrest (*n* = 1) three month after surgery or cervical/pulmonal metastatic tumor recurrence (*n* = 2) after 4 and 7 month. Intraoperative complications regarding graft osteotomy, assembly and fixation in recipient site did not occur.

Postoperative complications occurred in seven patients: Two fibula flaps were lost due to venous combustion in irradiated patients. Five patients suffered major complication (extensive wound dehiscence (*n* = 3) requiring secondary surgery, wound infection with loss of skin flap (*n* = 2), loss of transplant (*n* = 2), recurrence of tumor (*n* = 4; 3–10 month after histopathological R0-status; recurrence as cervical or pulmonal metastases), extensive bleeding requiring revision surgery (*n* = 1). Major complication arose only in patients with preoperative radiation. Minor complications arose in nine patients including partial (*n* = 6) or total loss of skin paddle (*n* = 1), limited wound dehiscence (self limiting by secondary healing) (*n* = 6) and venous obstruction with revision surgery (*n* = 2).

Analysis of workflow showed a planning period for reconstruction down to a minimum of 4 days (day of last necessary CT scan - mainly leg CT - to day of surgery). Mean time period was 8 days. Secondary reconstruction was not included in this analysis as the planning period could be chosen freely und thus was not shortened as much as possible.

### Costs were separated into personnel costs and material costs

In early planning a surgeon doing virtual workbench took 1 h per planning and laboratory work bench time of 1 h; and additionally a technician doing laboratory work of 4 h lead to personnel costs of 593€. Material costs for an in-house print of skull and fibula with 220€ and averaged laboratory material with 50€ for each planning session. This sums overall direct costs of 863€ for an in-house planning without the costs for the printer and the software.

In late planning the laboratory cost could be omitted. Planning time took in average 3 h. Planning was done completely done by a surgeon. Personnel cost were 367€. Material costs for surgical model and surgical guides were 150€. The overall costs for a complete in-house digital workflow thus were 517€.

## Discussion

Thirty patients were successfully planned through an in-house CAD/CAM algorithm for reconstruction with a fibula graft. Major complications did not occur in relation to the planning itself and was attributed to pre-radiated patients. Higher risk of complications and flap loss for this patient group are described in literature [20, 21]. As far this workflow solution was just used for fibula graft but could also be applied to other bone grafts like scapula or iliac crest flaps [6, 22]. In literature assessment of surgical time regarding CAD/CAM procedures is heterogenic but mostly states time reduction [7, 10, 23]. The percepted reduction of surgical time has not been tested here, but leads to a lower risk of general complications [11–13].

Work bench time was completely done by a surgeon. In our opinion the crucial virtual and laboratory work bench steps, like planning of the osteotomy lines, taking tumor resection and skin perforators in account, or the design of surgical templates need a specific background knowledge. This means these steps should be done by specific trained personnel and cannot be delegated. Thus the effect of reduction of surgery time is a result of a transition of manpower into the pre-surgical phase outside of the OR. The saved surgery time is thus only redeployed as already mentioned [7, 23]. Overall there is still an economization as only one person is needed for planning instead of a whole OR team.

An algorithm applicable without outsourcing makes CAD/CAM planning suitable even in urgent cases of primary cancer resections. One early shortcoming however of the current workflow is, that commercial software for surgery planning is available, but restricts rarely allows free data export thereby hindering a CAD/CAM pathway with internal resources. An open software or the development of such a solution with planning possibilities and appliances for template building does lead to further reduction of workbench time and further economization as show through the late developments in our workflow. This procedure may even allow the application in countries with lesser economic power inside the health system.

A complete in-house workflow proved more cost effective by reducing material costs, personnel costs and surgery times. A suitable outsourced 3D print costs about 700€. The self-printed 3D model costs approx. 150–220€ for each planning. In summary compared with literature estimations our in-house workflow (517–863€) seems to be lower than reported otherwise [24, 25]. The shortcoming of our economic evaluation is, that not all indirect costs (hard- and software) have been included and no calculation of the reduced intra-operative costs was possible. This question should be more clearly addressed in future studies including important direct and indirect costs.

A possible influence on flap survival resulting from the application of planning still needs evaluation. This far only the positive effect of pre-surgical planning on shape and esthetic outcome is described [7]. Our hypothesis is, that the extensive involvement of the surgeons with each case necessary during the planning phase might have a positive effect on flap survival and complication rate. However the follow up time in this case series is not sufficient for a profound answer.

## Conclusions

In conclusion it can be stated, that it is possible to apply a CAD/CAM workflow to fibula graft reconstruction within a few days making this technique available for immediate primary reconstruction of malignant tumors.

## Abbreviations

3D: Three dimensional
CAD: Computer aided design
CAM: Computer aided manufacture
CT: Computer tomography
DICOM: Digital imaging and communications in medicine
n: Number
